# Histiocitosis de células de Langerhans: reporte de caso y revisión de la literatura

**DOI:** 10.7705/biomedica.5430

**Published:** 2021-09-22

**Authors:** Miguel Ángel Medina, Wendy Meyer, Carolina Echeverri, Natalia Builes

**Affiliations:** 1 Grupo AUNA, Instituto de Cancerología Las Américas, Medellín, Colombia Instituto de Cancerología Las Américas Medellín Colombia; 2 Facultad de Medicina, Universidad Pontificia Bolivariana, Medellín, Colombia Universidad Pontificia Bolivariana Facultad de Medicina Universidad Pontificia Bolivariana Medellín Colombia; 3 Unidad de Cancerología, Hospital Pablo Tobón Uribe, Medellín, Colombia Unidad de Cancerología Hospital Pablo Tobón Uribe Medellín Colombia

**Keywords:** histiocitosis de células de Langerhans, histiocitosis, pediatría, conjuntivitis, dermatitis seborreica, Histiocytosis, Langerhans-cell, histiocytosis, pediatrics, conjunctivitis, dermatitis, seborrheic

## Abstract

La histiocitosis de células de Langerhans comprende un grupo heterogéneo de enfermedades inflamatorias cuyos principales componentes celulares son las células dendríticas y los macrófagos. El infiltrado inflamatorio puede afectar la piel y otros órganos, y el resultado clínico varía de leve a letal, dependiendo del subconjunto de células involucradas y el compromiso multisistémico. La demora en el diagnóstico puede ocurrir debido a su presentación inespecífica y a que los médicos tratantes no suelen sospecharla. Se reporta el caso de una lactante mayor a la cual, a pesar de múltiples consultas con síntomas inespecíficos pero característicos de la enfermedad, solamente se le pudo hacer el diagnóstico gracias a los hallazgos histopatológicos.

## Abstract (en)

Histiocytosis comprises a heterogeneous group of inflammatory diseases whose main cellular components are dendritic cells and macrophages. The inflammatory infiltrate can affect the skin and other organs and the clinical outcome varies from mild to fatal depending on the involved cell subset and multisystemic compromise. Delay in diagnosis may occur due to its non-specific presentation and to a low suspicion on the part of the clinician. We report the case of an infant who despite multiple consultations with nonspecific but characteristic symptoms of the disease was only finally diagnosed thanks to histopathological findings.

La histiocitosis de células de Langerhans es una alteración histiocítica poco frecuente que se caracteriza por la proliferación de células del sistema fagocítico mononuclear (monocitos, macrófagos, células dendríticas) en diferentes órganos y sistemas. Dicha proliferación puede ser localizada (lesión únicamente en la piel o una aislada en el hueso), o bien, generalizada en varios órganos y sistemas. Predomina en la infancia y en el sexo masculino, en una relación de 1,5:1 con el femenino, y afecta a 4 de 9 niños menores de 15 años por millón, con una edad media de presentación de 30 meses ^1^^-^^3^.

Su manifestación clínica es heterogénea y poco específica, lo que da lugar a múltiples cuadros clínicos, la mayoría de ellos con expresión cutánea precoz, rasgo que permite orientar el diagnóstico, pero también hay formas fulminantes asociadas con compromiso multisistémico, morbilidad aguda o crónica y mortalidad. Los pacientes se estratifican por riesgo según la extensión de la enfermedad y los órganos afectados ^4^. Los esquemas de estratificación de riesgo han cambiado en los últimos años y continúan evolucionando, pero son útiles para establecer el mejor esquema de tratamiento a partir del compromiso de cada paciente ^5^^,^^6^.

En Colombia, no se conoce con certeza la incidencia de esta enfermedad. En las series publicadas, no obstante, se evidencia un mayor compromiso sistémico, con una incidencia probablemente subestimada por la poca especificidad de los síntomas, y su asociación con el carácter crónico de la enfermedad y sus tratamientos. Es importante resaltar que el impacto de las características genéticas en la progresión y las secuelas a largo plazo es escaso. Asimismo, se ha señalado que las manifestaciones clínicas afectan el pronóstico, como en el caso de aquellas similares a las de la dermatitis seborreica, asociadas con el diagnóstico tardío, el consecuente aumento de la morbilidad y el empeoramiento del pronóstico ^7^^-^^9^.

En este contexto, se presenta el caso de una lactante con histiocitosis de células de Langerhans que inicialmente se diagnosticó erróneamente como dermatitis seborreica, y que presentaba un cuadro clínico crónico de lesiones en la piel que posteriormente comprometió múltiples sistemas. En el curso de la enfermedad, nunca se sospechó el diagnóstico de histiocitosis de células de Langerhans, el cual finalmente se precisó de forma incidental mediante el estudio patológico.

## Presentación del caso

Se trata de una niña lactante de 12 meses de edad, sin antecedentes personales ni familiares de importancia. A los seis meses de edad, presentó erupción micropapular, eritematosa, puntiforme y pruriginosa en los hombros y el cuello. Posteriormente, aparecieron lesiones descamativas en cuero cabelludo y tórax indicativas de dermatitis seborreica. Recibió múltiples tratamientos hidratantes durante seis meses sin ninguna mejoría.

A los 12 meses, de forma súbita y con rápida evolución, presentó edema palpebral izquierdo asociado con eritema periorbitario, pero sin otros síntomas. En el examen clínico, la paciente presentaba aumento de volumen en el tercio medio de la hemicara izquierda, con importante asimetría, y el párpado inferior izquierdo tenía coloración violácea.

En el hemograma inicial se reportó: 10 g/dl de hemoglobina; 32,1 % de hematocrito; 14.700 mm^3^ leucocitos; 46 % de neutrófilos; 46,4 % linfocitos; 594.000 mm3 plaquetas, y 2 mg/dl de proteína C reactiva. La función hepática y la renal eran normales para su edad.

**Figura 1:**
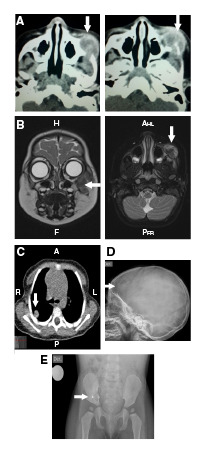
A. Tomografia inicial con contraste de órbita simple: se observa una lesión isodensa encapsulada en la región infraorbitaria izquierda que muestra cápsula que capta el medio de contraste. El centro de la lesión es el cuerpo del hueso malar izquierdo que está erosionado y remodelado por la lesión. La lesión erosiona el arco cigomático izquierdo, se extiende a la órbita izquierda y rechaza el globo ocular en sentido central y superior. Sus diámetros son: cefalo-caudal de 2,3 cm, antero-posteriorde 2,2 cm y transverso de 2 cm. B. Resonancia magnética con contraste de cara y cráneo: se aprecia una lesión tumoral en las regiones infraorbitaria y maxilar izquierdas, con compromiso de tejidos blandos e infiltración ósea de maxilar, arco cigomático y piso orbitario, y con extensión infraorbitaria y al espacio masticatorio; se observa una lesión que compromete la tabla ósea frontal derecha. C. Tomografia computarizada simple de tórax: nodulos pulmonares en la periferia del pulmón derecho. D. Radiografía simple de cráneo: lesión lítica de bordes esclerosos y 23 x 15 mm en el hueso frontal derecho. E. Radiografía de pelvis: lesión lítica de bordes esclerosos en de la porción inferior del ilíaco derecho, adyacente a la articulación sacroilíaca, de aproximadamente 25 x 15 mm.

En la tomografía computarizada (TC) de órbita simple y con contraste, se observó una lesión isodensa encapsulada en la región infraorbitaria izquierda (figura 1A). La resonancia magnética (RM) de cráneo, órbita y cuello reveló una lesión tumoral en la región infraorbitaria y la maxilar izquierdas, con compromiso de tejidos blandos e infiltración ósea del maxilar, el arco cigomático y el piso orbitario, con extensión infraorbitaria y extensión al espacio masticatorio, y lesiones nodulares que comprometían la glándula parótida izquierda y la tabla ósea frontal derecha (figura 1B).

En la TC simple de tórax, se observaron cuatro nódulos pulmonares alojados en la periferia del pulmón derecho y compromiso de la columna vertebral en T4 (figura 1C). La paciente nunca presentó síntomas respiratorios y, dada su edad, no fue posible practicar pruebas de función pulmonar. En la radiografía de serie ósea se encontraron lesiones líticas en cráneo y pelvis, pero no así en los huesos largos (figura 1D).

**Figura 2:**
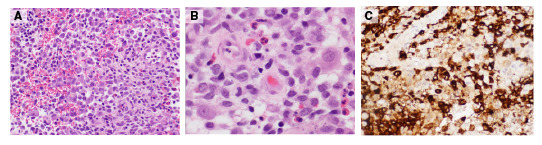
En los cortes se observan fragmentos de tejido infiltrado por una proliferación de células elongadas con hendiduras nucleares. En el fondo encuentran células gigantes multinucleadas y un menor número de eosinófilos. A. Hematoxilina-eosina, 40X. B. Hematoxilina-eosina, 100 X. C. CD1a

Se hizo una biopsia por escisión de la lesión infrapalpebral izquierda, en la que se encontraron fragmentos de tejido infiltrado por proliferación de células elongadas con hendiduras nucleares. En el fondo, se observaron células gigantes multinucleadas y un menor número de eosinófilos (figura 2). Los exámenes inmunohistoquímicos específicos para CD1 (tinción de membrana citoplasmática), proteína S100 (tinción nuclear y citoplasmática) y CD68 (marcador histiocítico) fueron positivos. La biopsia permitió confirmar el diagnóstico histopatológico de histiocitosis de células de Langerhans.

Por los órganos afectados y la extensión de la enfermedad, se hizo una estratificación del riesgo y se la clasificó como una enfermedad multisistémica sin compromiso de órgano de riesgo. Se inició el tratamiento según el protocolo LCH III de la Sociedad Internacional de Histiocitosis, el cual incluye el uso de vinblastina durante seis semanas y de prednisona durante cuatro semanas. La mejoría clínica y radiológica en esta paciente se valoró como intermedia, por lo que se repitió el tratamiento. Al final de las 12 semanas de vinblastina, hubo resolución de las lesiones, por lo cual se continuó con un tratamiento de mantenimiento durante 12 meses. Al cabo de 48 meses de seguimiento, la paciente no presentaba signos de recaída de la enfermedad.

## Discusión

La histiocitosis de células de Langerhans es una afección poco común que se presenta principalmente en la infancia y con diversas formas clínicas. Existe controversia sobre la etiología de la enfermedad y se ha debatido por años si se trata de una alteración inmunitaria o una neoplasia.

Dado que puede aparecer como una lesión de resolución espontánea o con tratamiento antiinflamatorio, se la ha considerado un proceso inflamatorio. Sin embargo, en otros casos se puede manifestar con compromiso diseminado y requerir tratamientos más agresivos, incluida la quimioterapia, lo cual sugiere una posible naturaleza neoplásica ^10^^-^^12^, ya que se encuentran activas las células de Langerhans patológicas, así como citocinas inflamatorias que expresan la CD40.

La clonalidad de las células de Langerhans era la teoría que apoyaba un origen neoplásico, pero la clonalidad en células inmunitarias no es suficiente para clasificar una enfermedad como maligna ^13^. En el 2010, se identificó una serie de anomalías genéticas involucradas, y la Organización Mundial de la Salud (OMS) consideró la enfermedad como alteración genética neoplásica, ya que corresponde a una proliferación neoplásica clonal de las células de Langerhans. Se ha reportado la mutación V600E en el gen *BRAF,* la cual interviene en la vía de activación de las cinasas MAPK, frecuentemente mutada en algunas neoplasias, y se encarga de enlazar las señales extracelulares con la maquinaria intracelular necesaria para controlar el crecimiento, la proliferación y la diferenciación celulares ^10^. Estas observaciones sugieren el origen neoplásico de la enfermedad ^14^.

En la actual clasificación de las alteraciones histiocíticas, propuesta por la Sociedad Internacional de Histiocitosis en el 2016, los pacientes son clasificados en cinco grupos con base en aspectos clínicos, histológicos y moleculares: grupo l, o Langerhans; grupo C, cutáneo y mucocutáneo; grupo M, histiocitosis maligna; grupo R, enfermedad de Rosai Dorfman, y grupo H, linfohistiocitosis hemofagocítica y síndrome de activación del macrófago. La histiocitosis de células de Langerhans corresponde al grupo l o Langerhans ^15^.

### Presentación clínica

Por lo general, la enfermedad tiene una presentación clínica heterogénea, que va desde la forma de resolución espontánea hasta el compromiso multisistémico, cuyo curso es impredecible. Se puede presentar regresión espontánea, así como recurrencias crónicas y deterioro que pueden llegar a ser fatales ^16^. Su clasificación corresponde al número de lesiones, sitios involucrados y extensión. Estos criterios, sumados a la reacción al tratamiento, son una herramienta valiosa para determinar el riesgo de mortalidad.

El sistema más comúnmente afectado es el óseo (80 %), principalmente los huesos del cráneo, seguidos por los huesos largos y las vértebras ^17^^,^^18^^)^ . El compromiso de la piel puede verse hasta en una tercera parte de los pacientes y afecta con mayor frecuencia las áreas intertriginosas. Las formas de presentación comúnmente descritas son lesiones en forma de erupción papular, ulcerativas o vesiculares, úlceras hipopigmentadas que posteriormente sanan, así como formas similares a la dermatitis seborreica ^10^^,^^19^ . Poompuen, *et al.,* han señalado cómo la similitud con la dermatitis seborreica puede retrasar el diagnóstico por la dificultad de distinguir las lesiones de una y otra condición y, además, porque la dermatitis seborreica es una entidad comúnmente observada en la edad pediátrica, como en el presente caso. Las lesiones óseas asociadas con la dermatitis seborreica pueden ser la clave para una detección temprana de la histiocitosis de células de Langerhans ^8^, la cual puede sospecharse ante esta asociación y la poca mejoría con los tratamientos instaurados.

Además, se ha descrito el compromiso periorbitario (proptosis), aunque la afectación del nervio óptico no es clara ^20^. Las cadenas ganglionares cervicales se ven comprometidas y alcanzan tamaños muy grandes, pudiendo afectarse también ganglios del mediastino y el abdomen. No se han descrito casos de afectación en gónadas, riñón o vejiga.

El compromiso de los huesos del cráneo y la región hipotalámica es frecuente, ocasionando diabetes insípida (50 %). El compromiso de la hipófisis anterior, acompañada de deficiencia de la hormona del crecimiento, es la segunda endocrinopatía más frecuente (10 %) y hasta el 60 % de los pacientes presenta hipotiroidismo, hipogonadismo e hiperprolactinemia cuando hay este compromiso hipofisiario ^21^.

En el sistema nervioso central, se presenta como una enfermedad neurodegenerativa con manifestaciones progresivas: síndrome cerebeloso (temblor, anormalidades en reflejos, ataxia, dismetría) o compromiso motor, parálisis pseudobulbar, disartria, disfagia, espasticidad, problemas de aprendizaje o, incluso, enfermedades psiquiátricas. También, se han descrito lesiones tumorales intracraneales que se manifiestan como un síndrome paraneoplásico autoinmunitario ^16^. Cuanto mayor sea el compromiso de los huesos del cráneo y del sistema neuroendocrino, mayor será el riesgo de enfermedad neurodegenerativa ^21^^-^^23^.

También, puede presentarse compromiso de órganos como hígado, bazo, médula ósea y ganglios linfáticos; además, puede haber un mayor riesgo de mortalidad al afectarse ciertos órganos, principalmente, el sistema hematopoyético, el hígado y el bazo ^13^^,^^24^. Por otra parte, la histiocitosis pulmonar de células de Langerhans es frecuente en adultos, especialmente en fumadores. En niños, este tipo de presentación es poco frecuente y, generalmente, se asocia con compromiso multisistémico extenso y mayor morbilidad ^25^.

### Diagnóstico

El diagnóstico se basa en hallazgos clínicos, radiográficos e histopatológicos mediante biopsia por escisión, en la cual se evidencia la infiltración de tejidos por histiocitos que expresan la CD207 y la CD1+ en la superficie. A pesar de ser un diagnóstico relativamente fácil de confirmar, muchas veces se atrasa por falta de sospecha clínica, como en el caso de la paciente que se reporta ^13^^,^^24^.

Se debe evaluar la extensión de la enfermedad con radiografías del cráneo, el esqueleto y el tórax. En el hemograma completo se suele observar anemia leve, y puede presentarse pancitopenia por disfunción de la médula ósea asociada con hepatoesplenomegalia. En el aspirado de médula ósea inicial, la celularidad es normal, pero una vez avanzada la enfermedad, puede tornarse hipocelular y es posible ver células de Langerhans y macrófagos hemofagocitados. Se debe valorar la función hepática (aminotransferasas, bilirrubinas, gamma-glutamil transferasa) y la renal para descartar compromiso en estos órganos ^20^^,^^24^.

La ecografía de abdomen puede ayudar a descartar compromiso hepático, en tanto que la tomografía de cráneo permite evaluar lesiones en órbita, mastoides, esfenoides y huesos temporales. La resonancia magnética puede evidenciar lesiones histiocíticas ocupantes de espacio en los hemisferios cerebrales, la hipófisis, las meninges, el plexo coroideo, el tallo cerebral y la médula espinal, que podrían tomarse por tumores del sistema nervioso central, metástasis o enfermedades granulomatosas, en tanto que las lesiones degenerativas se podrían interpretar como áreas de atrofia cerebral ^24^. Los estudios de imágenes pueden ser de utilidad para evaluar la actividad de la enfermedad y, según el tejido bajo estudio, para establecer la necesidad de recurrir a la resonancia magnética, la tomografía por emisión de positrones o la TC, siendo esta última la mejor para evaluar los huesos del cráneo, el parénquima cerebral y las lesiones en la hipófisis ^24^^,^^26^.

## Conclusiones

La histiocitosis de células de Langerhans en una enfermedad poco común, cuyo pronóstico y resultado están determinados por el diagnóstico temprano y el consecuente tratamiento oportuno. Por ello, es de vital importancia considerarla entre los diagnósticos diferenciales de las lesiones de piel que no mejoran con los tratamientos instaurados y para descartar enfermedades comunes según el grupo de edad, punto clave para el médico general y el pediatra, con el fin de remitir a los pacientes al especialista para un pronto manejo que disminuya las complicaciones a largo plazo.

Se requieren más estudios para conocer mejor su fisiopatología en aras de lograr un diagnóstico temprano, de manera que no haya que esperar la evolución de la enfermedad hasta la invasión sistémica. Como se sabe, enfermedad que no se conoce, no se diagnóstica.
